# ﻿Two new Parahiraciini planthoppers from Central Vietnam in the genera *Gelastyrella* and *Pseudochoutagus* (Hemiptera, Fulgoromorpha, Issidae)

**DOI:** 10.3897/zookeys.1257.155185

**Published:** 2025-10-24

**Authors:** Jérôme Constant, Thai-Hong Pham, Hoai Thu Thi Nguyen

**Affiliations:** 1 Royal Belgian Institute of Natural Sciences, O.D. Taxonomy & Phylogeny - Entomology, Vautier street 29, B-1000 Brussels, Belgium Royal Belgian Institute of Natural Sciences Brussels Belgium; 2 Mientrung Institute for Scientific Research, Vietnam National Museum of Nature, VAST, 321 Huynh Thuc Khang, Hue, Vietnam Mientrung Institute for Scientific Research, Vietnam National Museum of Nature Hue Vietnam; 3 Graduate School of Science and Technology, Vietnam Academy of Science and Technology, 18 Hoang Quoc Viet, Hanoi, Vietnam Vietnam Academy of Science and Technology Hanoi Vietnam; 4 Vietnam National Museum of Nature, Vietnam Academy of Science and Technology (VAST), 18 Hoang Quoc Viet, Hanoi, Vietnam Vietnam National Museum of Nature, Vietnam Academy of Science and Technology Hanoi Vietnam

**Keywords:** Biodiversity, Fulgoroidea, Indochina, taxonomy

## Abstract

Two new planthopper species of the family Issidae, tribe Parahiraciini, are described from Central Vietnam: *Gelastyrella vuquangensis***sp. nov.** from Vu Quang National Park in Ha Tinh Province, and *Pseudochoutagus nuichuanus***sp. nov.** from Nui Chua National Park in Khanh Hoa Province. Illustrations of habitus and terminalia of the new species are given, as well as a distribution map and photographs of live specimens and their habitat.

## ﻿Introduction

With more than 1,100 species in about 230 genera (Bourgoin 2025), the family Issidae Spinola, 1839 is a large family of planthoppers (Hemiptera, Fulgoromorpha) with a worldwide distribution. The family currently represents about 8% of the species of Fulgoromorpha. Despite recent progress in the documentation of new taxa, the fauna of some major regions such as tropical Africa, New Guinea and Australia, remain very poorly documented (Gnezdilov and Fletcher 2010; Gnezdilov 2013; Gnezdilov et al. 2022; Constant and Semeraro 2023).

The tribe Parahiraciini currently counts 23 species in 13 genera (Constant and Pham 2024a, 2024b) in Vietnam, and several species were described or recorded from North and Central Vietnam in recent years (Constant and Pham 2024a, 2024b).

Our study of Issidae in the collections of the Vietnam National Museum of Nature and the Royal Belgian Institute of Natural Sciences revealed two new species of Parahiraciini from Central Vietnam, in the genera *Gelastyrella* Yang, 1994 and *Pseudochoutagus* Che, Zhang & Wang, 2011. The present paper aims to describe these two new species and provide some information on their habitat and biology, as a new contribution to the Vietnamese issid fauna.

## ﻿Materials and methods

The specimens were captured by hand using small transparent vials with which they were slowly covered or by sweeping the lower vegetation, bushes and lower branches of trees along forest trails.

The photographs of habitats and live specimens were taken with an Olympus Tough 6 camera; some specimens were placed in a fine mesh cage when necessary but in this case, it is mentioned in the caption. The collected specimens were photographed with a Leica EZ4W stereomicroscope with integrated camera, and the images were stacked with CombineZ software and optimized with Adobe Photoshop CS3; all photographs are by JC. The distribution maps were produced with SimpleMappr (Shorthouse 2010). The genitalia were extracted after soaking the abdomen in a 10% solution of potassium hydroxide (KOH) at room temperature for about 12 hours. The pygofer was separated from the abdomen and the aedeagus dissected with a needle blade for examination. The whole was thoroughly rinsed in 70% ethanol, then placed in glycerine for preservation in a tube attached to the pin of the corresponding specimen. The hind wings were glued with white glue on a small white cardboard rectangle attached to the pin of the corresponding specimen.

The external morphological terminology follows O’Brien and Wilson (1985) and for the terminalia, Bourgoin and Huang (1990), Gnezdilov (2003), and Gnezdilov et al. (2014b). The metatibiotarsal formula gives the number of spines on (side of metatibia) apex of metatibia / apex of first metatarsus / apex of second metatarsus. The terminology of the wing venation follows Bourgoin et al. (2015). The higher classification follows the most recent one as published by Gnezdilov et al. (2022).

The measurements were taken as in Constant (2004) and the following abbreviations are used:

**BB** maximum breadth of the body

**BF** maximum breadth of the frons

**BTg** maximum breadth of the tegmen

**BV** maximum breadth of the vertex

**BW** maximum breadth of the hind wing

**LF** length of the frons at median line

**LT** total length (apex of head to apex of tegmina)

**LTg** length of the tegmen

**LV** length of the vertex at median line

**LW** maximum length of the hind wing

Abbreviations used for the collections:

**RBINS** Royal Belgian Institute of Natural Sciences, Brussels, Belgium

**VNMN** Vietnam National Museum of Nature, Hanoi, Vietnam

Other abbreviation:

**ss** sensu stricto

## ﻿Taxonomy

### ﻿Family Issidae Spinola, 1839

**Subfamily Issinae Spinola, 1839**

**Tribe Parahiraciini Cheng & Yang, 1991**

#### ﻿Subtribe Parahiraciina

Taxon classificationAnimaliaHemipteraIssidae

Cheng & Yang, 1991

FB3C3BA3-A7C6-511C-8721-C19D9AA03F82

##### Type genus.

*Parahiracia* Ôuchi, 1940 (junior synonym of *Fortunia* Distant, 1909).

#### ﻿Genus Gelastyrella

Taxon classificationAnimaliaHemipteraIssidae

Yang, 1994

8705136F-DD47-5DDE-9570-168C8C37F0B9

Gelastyrella Yang, 1994 in Chan & Yang, 1994: 90. Type species: Gelastyrella litaoensis Yang, 1994, by original designation.

##### Diagnosis.

The genus *Gelastyrella* can be differentiated from the other genera of Parahiraciina by the following combination of characters (based on Zhang et al. 2020):

1. the frons about as long a wide, with median carina and a transverse carina under the dorsal margin;
2. the tegmina tapering towards apex, with vein ScP+R forked near base, MP 3-branched with first fork around midlength and CuA unforked;
3. the tri-lobed hind wings (with A2 lobe reduced);
4. the first metatarsomere with a dense pad of spines ventrally, in addition to spines of the apical margin;
5. the ventral lobe of the periandrium strongly expanded basiventrad in rounded lobes;
6. the aedeagus with one pair of lateroventral processes and well developed suspensorium.

##### Note.

According to Zhang et al. (2020), the genus *Gelastyrella* differs from the genus *Thabena* Stål, 1866 by the male genitalia with a well-developed suspensorium (suspensorium small in *Thabena*), and the ventral lobe of the periandrium strongly convex in lateral view (straight in *Thabena*). The two genera had been previously synonymized by Gnezdilov (2009, 2015), but we follow here the most recent classification. Several undescribed species from South-East Asia are present in the collections (Constant and Pham unpublished), which, together with additional molecular data, could help to refine the status of these genera in the future.

##### Species included (distribution).

*Gelastyrella litaoensis* Yang, 1994 (China: Hainan, Fujian, Guangxi; Taiwan; Vietnam: Hoa Binh, Ninh Binh, Quang Tri, Thanh Hoa and Thừa Thiên-Huế provinces – Chan and Yang 1994; Gnezdilov et al. 2014a; Gnezdilov 2015; Constant and Pham 2024b).

*Gelastyrella vuquangensis* sp. nov. (Vietnam: Ha Tinh Province).

#### ﻿ Gelastyrella vuquangensis sp. nov.

Taxon classificationAnimaliaHemipteraIssidae

2A3FFD6C-F06C-5DFA-8EBD-252EC091C284

https://zoobank.org/4D5481ED-2F5B-43B2-8812-44449E0E653D

Figs 1, 2, 3, 4, 5, 6, 7, 8

##### Type material.

***Holotype*** ♂, Vietnam • Ha Tinh Province, Vu Quang National Park., near Khe Chè station; 18°22'38"N, 105°18'41"E; 13–15 Jul. 2023; J. Constant, J. Bresseel and L. Semeraro leg.; I.G.: 34.661; RBINS.

***Paratypes***, Vietnam • 8 ♂♂, 15 ♀♀; same data as for holotype; RBINS • 8 ♂♂, 2 ♀♀; Ha Tinh Province, Vu Quang National Park, near Khe Chè station; 18°22'38"N, 105°18'41"E; 13–15 Jul. 2023; Trung T. Vu and Hoai T.T. Nguyen leg.; VNMN-E. 000.016.100-VNMN-E.000.016.109; VNMN.

##### Diagnosis.

The species is externally rather similar to *G. litaoensis* Yang, 1994 (see illustrations in Constant and Pham 2024b: figs 19–21) but it is shorter (on average, ♂: 6.8 mm, ♀: 7.4) than the Vietnamese specimens of *G. litaoensis* (on average, ♂: 8.1 mm, ♀: 8.9), and it is generally paler than *G. litaoensis*; it shows gonostyli with a short posterior lobe and a short, wide capitulum in lateral view (lobe and capitulum elongate in *G. litaoensis*); the periandrium of the male shows moderately developed basiventral lobes (lobes massive, strongly projecting anteriad in *G. litaoensis*); the lateroventral processes of the aedeagus are strongly curved mesad in distal portion, ventrally overlapping (more or less parallel in *G. litaoensis*); the process of sternite VII of females is apically emarginate in middle (rounded in *G. litaoensis*).

##### Description.

***Measurements and ratios***: LT: ♂ (*n* = 9): 6.8 mm (6.6–7.2); ♀ (*n* = 9): 7.4 (7.2–7.5). LT/BB = 1.6; LTg/BTg = 2.1; LW/BW = 1.5; LV/BV = 0.5; LF/BF = 1.0.

**Figure 1.**
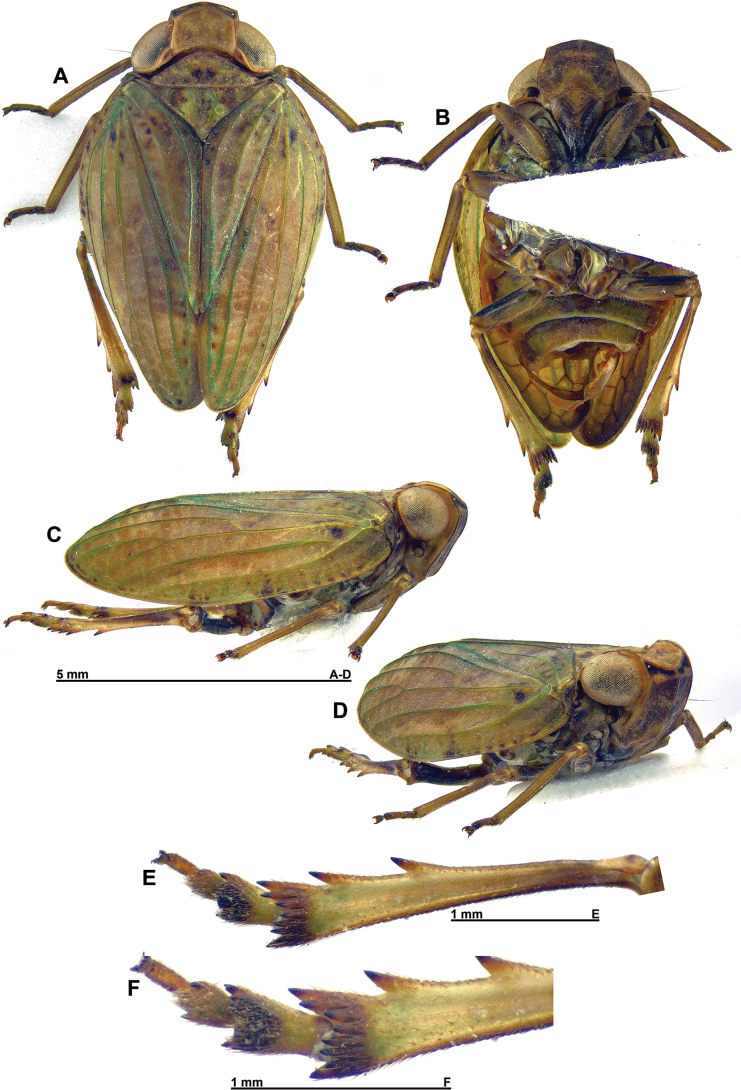
*Gelastyrella vuquangensis* sp. nov., holotype ♂ (RBINS). A. Habitus, dorsal; B. Habitus, ventral; C. Habitus, lateral; D. Habitus, anterolateral; E. Metatibia and metatarsus, ventral; F. Distal portion of metatibia and metatarsus, ventral view.

**Figure 2.**
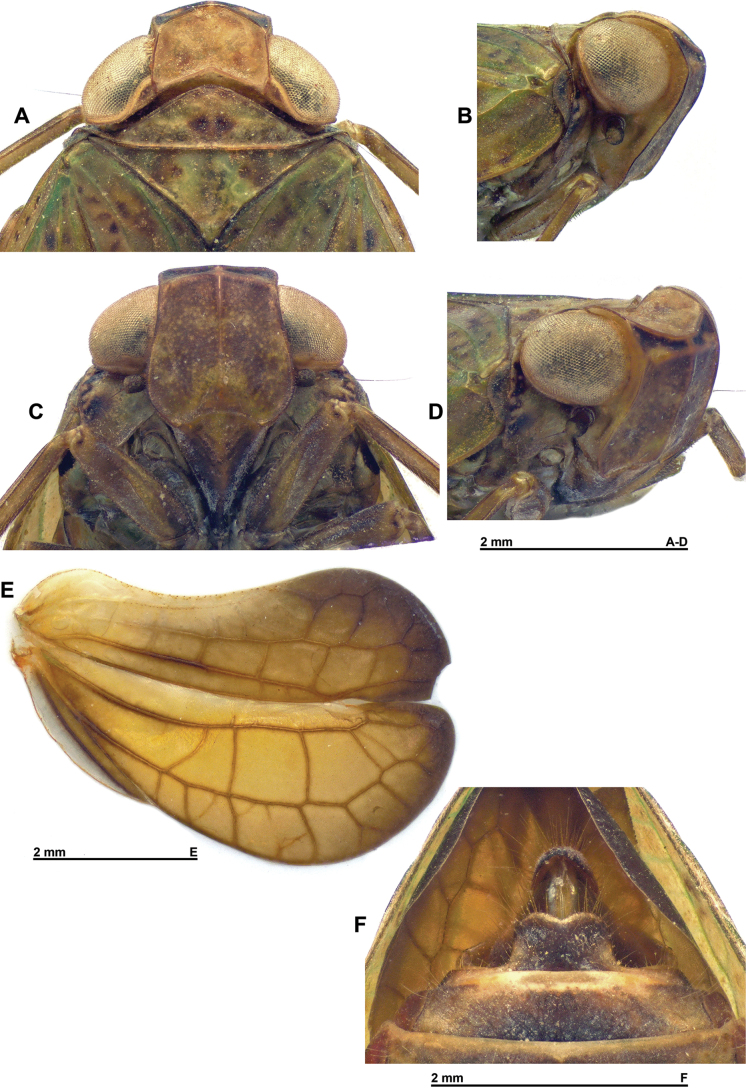
*Gelastyrella vuquangensis* sp. nov. A–E. Holotype ♂ (RBINS). A. Head and thorax, dorsal; B. Head and thorax, lateral; C. Frons, perpendicular; D. Head and thorax, anterolateral; E. Right hind wing; F. Paratype ♀ (RBINS), terminalia.

***Head*** (Figs 1A–D, 2A–D): vertex variegated brown, about twice as broad as long in midline, with all margins carinate; anterior and posterior margins distinctly curved, subparallel; lateral margins weakly curved, subparallel; disc excavate with obsolete median carina. Side of head yellowish with darker area between anteroventral portion of eye and anterior margin; anteroventral angle weakly projecting anteriorly. Frons appearing weakly elongate and generally smooth, variegated brown (darker than vertex) with blackish dorsal line between dorsal margin and distinct subdorsal transverse carina; median carina extending from dorsal margin down to rounded frontoclypeal suture; dorsal margin concave, lateral margins distinctly sinuate. Clypeus variegated dark brown with paler median line (more or less distinct), weakly elevated medially. Labium brown with last segment longer than broad, and shorter than penultimate. Antennae dark brown; scape short, ring-shaped; pedicel subcylindrical.

***Thorax*** (Figs 1A, C, D, 2A–D): pronotum variegated yellowish to greenish brown; about 0.6 times as long as mesonotum in midline; anterior margin carinate, moderately, roundly protruding anteriorly between eyes; posterior margin curved, raised; no median carina but with impressed point on each side of median line; lateral portion behind eye very narrow, laminate; blunt, pale yellowish tubercles scattered on outer margin of paranotal lobes; paranotal lobes broad, brown in outer portion, pale yellowish in inner portion and with darker marking under eye, and with posteroventral angle rounded. Mesonotum variegated yellowish to or greenish brown without distinct carinae; smooth, slightly depressed before scutellum. Tegulae yellowish to greenish brown.

***Tegmina*** (Figs 1A–D, 6): variegated yellowish to or greenish brown with irregular brown markings (more developed in females), usually covered in fine pruinous wax in live specimens; subcoriaceous with longitudinal veins slightly darker than background, elevated and with a dense reticulum of weak pale yellow veinlets; shape elongate and convex, about 2.1 times as long as broad with sides broadly rounded, widest at basal 1/3; tapering towards narrowly rounded apex. Postclaval margin nearly straight on most length, weakly notched at apex of clavus. Epipleuron present, moderate. Clavus closed, reaching 2/3 of length of tegmen.

Venation: ScP+R forking close to base after rather short common stem, ScP+RA and RP running more or less parallel to costal margin and not forked; MP forked after moderate common stem, resulting vein MP_1_ forked again further; CuA simple, sinuate, more or less parallel to claval joint, then to sutural margin and merging with latter before apex of tegmen; Pcu and A1 fused at slightly beyond 2/3 of clavus, resulting Pcu+A1 reaching apex of clavus.

***Hind wings*** (Fig. 2E): yellow-brown with distal portion of lobes Sc-R-MP-CuA and CuP-Pcu-A1, and lobe A2 darker brown; venation darker than corresponding background; wing deeply bilobed at CuP; costal margin distinctly bisinuate; CuP-Pcu-A1 lobe broadly rounded along postclaval margin and about as wide as Sc-R-MP-CuA lobe; both lobes approximately the same length, angularly rounded at apex; A2 lobe narrow. Venation: longitudinal veins ScP-R-MP-Cu well distinct; Pcu and A1 separated; numerous cross-veins; cross-veins delimiting large cells between Pcu and A1; A2 lobe narrow.

***Legs*** (Fig. 1A–F): rather elongate and slender, yellowish to greenish brown with metafemora, pro- and mesotarsi, and spines of hind legs, dark brown; posterior margin of pro- and mesofemora with row of acute minute teeth. Metatibiae with 2 lateral spines in distal half and 8 apical spines. First segment of metatarsus with strong tooth on each side, and large ventral pad of microspines in distal portion. Metatibiotarsal formula: (2) 8 / 2++ / 2.

***Abdomen*** (Fig. 1B): yellowish to greenish with wide, darker band in middle.

**Figure 3.**
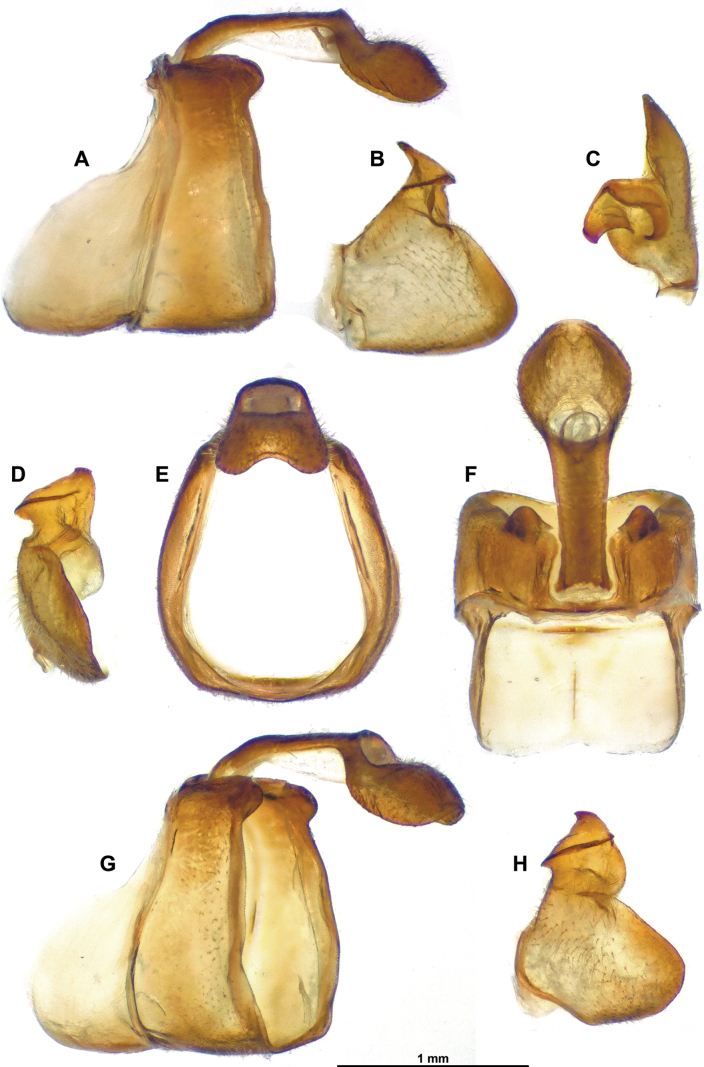
*Gelastyrella vuquangensis* sp. nov., holotype ♂ (RBINS). A. Pygofer and anal tube, lateral; B. Gonostylus, lateral; C. Idem, dorsal; D. Idem, caudal; E. Pygofer and anal tube, caudal; F. Idem, dorsal; G. Idem, posterolateral; H. Gonostylus, posterolateral.

**Figure 4.**
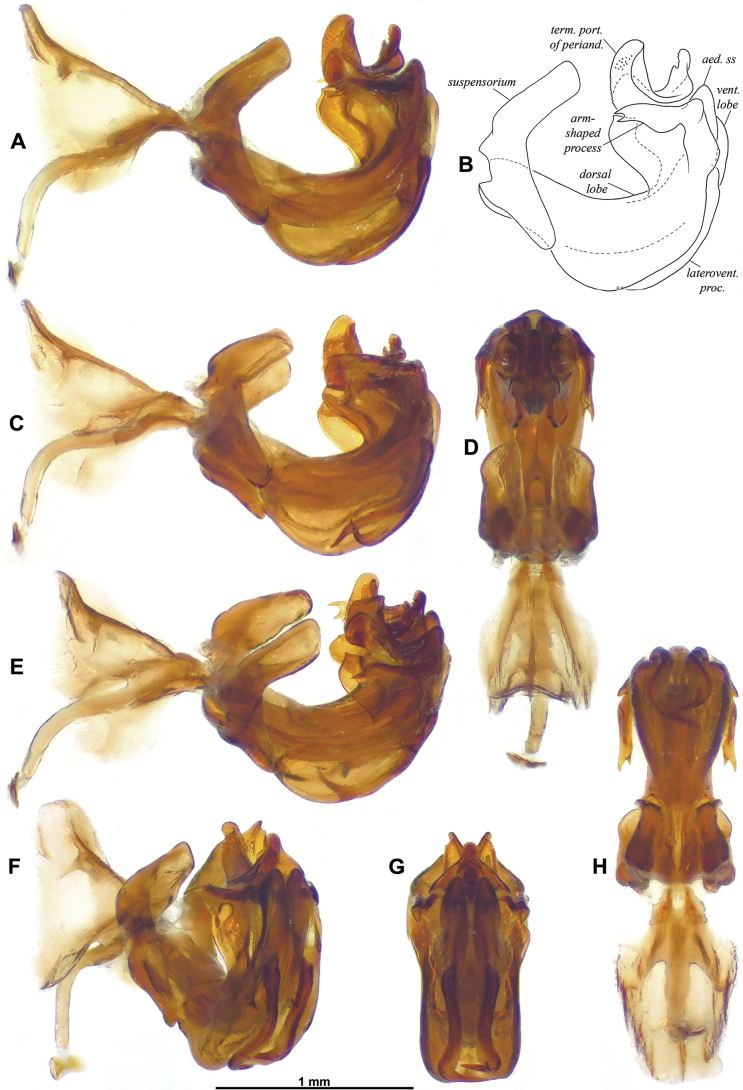
*Gelastyrella vuquangensis* sp. nov., holotype ♂ (RBINS), aedeagus. A, B. Lateral view; C. Lateroventral view; D. Dorsal view; E. Laterodorsal view; F. Laterocaudal view; G. Caudal view; H. Ventral view.

**Figure 5.**
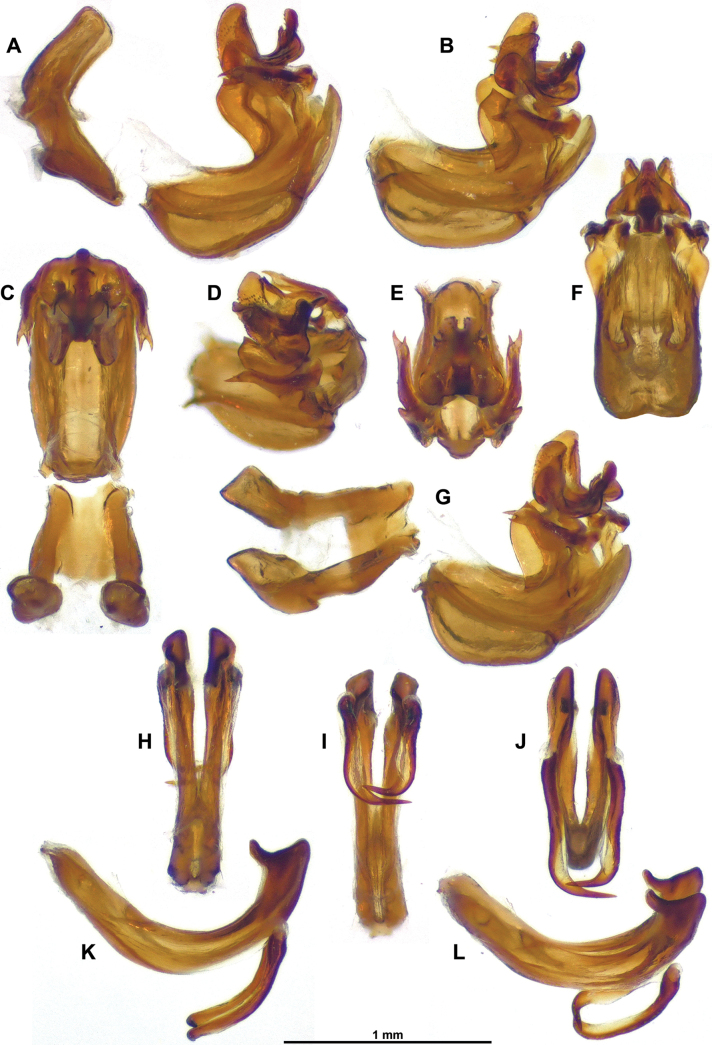
*Gelastyrella vuquangensis* sp. nov., holotype ♂ (RBINS). A–G. Periandrium; A. Lateral view (with suspensorium); B. Laterodorsal view; C. Dorsal view (with suspensorium); D. Latero-posterodorsal view; E. Posterodorsal view; F. Caudal view; G. Laterocaudal view (with suspensorium); H–L. Aedeagus ss; H. Dorsal view; I. Ventral view; J. Posteroventral view; K. Lateral view; L. Laterodorsal view.

***Terminalia*** ♂ (Figs 3–5): pygofer (Fig. 3A, E–G) about 2.0 times as high as long in lateral view (widest in ventral portion), with anterior margin incurved in dorsal portion and posterior margin more or less straight up to dorsal lobe directed caudad; posterior margin dorsally deeply excavate (U-shaped excavation wider towards base). Gonostyli (Fig. 3B–D, H) in lateral view, rather short and robust, subtriangular, produced caudad in a short lobe rounded apically, and moderately concave; capitulum massive, with rather short and wide neck, spiralling up with lateral, more or less oblique, laminate projection, and apex ended in oblique lamina directed cephalo-dorsomesad. Anal tube (Fig. 3A, E–G) racket-shaped in dorsal view, more or less dorsoventrally flattened, with basal narrow shaft representing 3/5 of length, nearly 2.4 times as long in midline as wide (widest point in distal portion); distal rounded portion starting at anal opening, convex with all margins downcurved; apical margin with round ventral emargination in caudal view. Aedeagus (Figs 4, 5) symmetrical, strongly recurved dorsad (in lateral view), with massive suspensorium consisting of large, boomerang-shaped lateral parts linked by wide, flat connective in distal portion (connective placed under aedeagus ss). Periandrium (Figs 4, 5A–G) complex, basally with moderately developed lobes projecting ventrad, ventral lobe rather simple, apically rounded, shorter than dorsal lobe; dorsal lobe in distal portion with lateral (external) arm-shaped processes directed cephalad and apically bifid, mesolateral laminate processes projecting cephalad with anterior margin sinuate and dorsal angle pointed; terminal portion mobile, articulated, at rest covering subapical processes of aedeagus ss, strongly curved in lateral view, anterior portion with two oblique laminae projecting laterodorsad (small teeth on inner wall), posterior portion upcurved, ended in elongate, apically rounded process (in lateral aspect), subtriangular in caudal view. Aedeagus ss (Fig. 5H–L) elongate, curved in lateral view, bifid along distal 2/3; subapical strong, upcurved dorsal process on each shaft, slightly directed mesad in dorsal view; lateroventral processes strong, directed anteroventrad in lateral view; in (postero)ventral view, sinuate in basal portion, then strongly curved mesad (rounded right angle) and tapering to narrow point (distal portion overlapping ventrally). Connective (Fig. 4A–F) well developed, elongate, curved, with massive tectiductus, more or less compressed laterally with large foramen.

***Terminalia*** ♀ (Fig. 2F): hind margin of sternum VII with large process directed caudad, with lateral margins curved and posterior margin distinctly emarginate in middle.

##### Etymology.

The species epithet refers to Vu Quang National Park in Ha Tinh Province, where the new species was discovered.

##### Biology.

**Figure 6.**
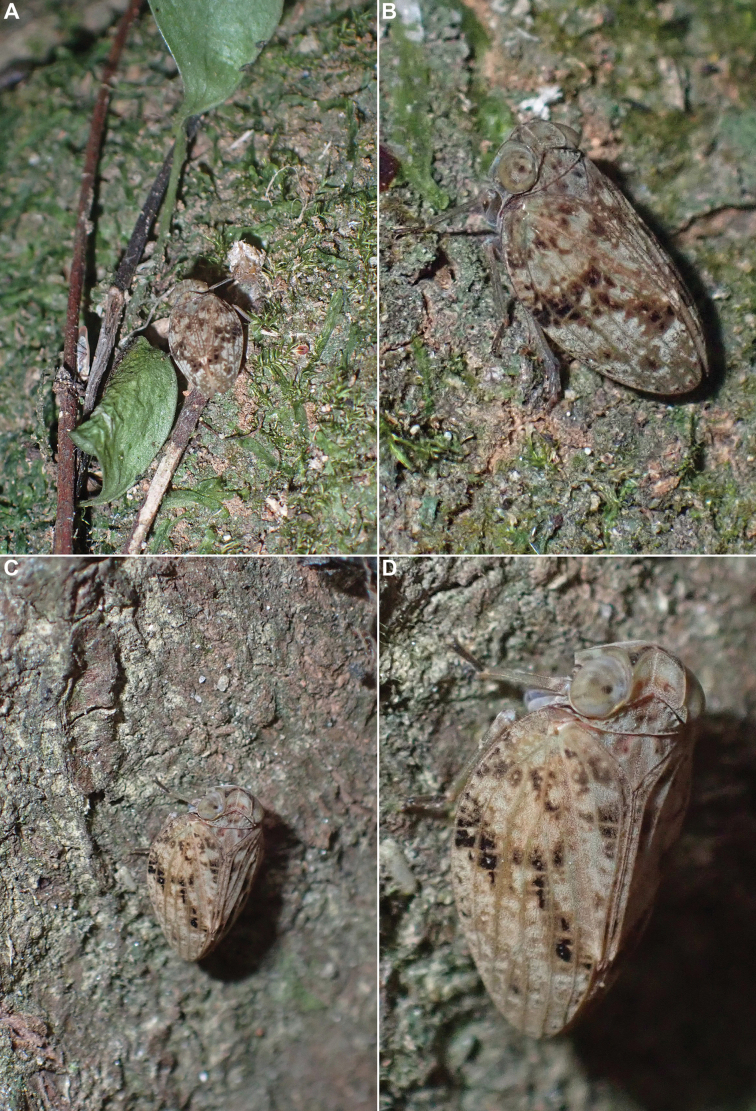
*Gelastyrella vuquangensis* sp. nov. A–D. Live specimens in Vu Quang National Park, 14 July 2023.

**Figure 7.**
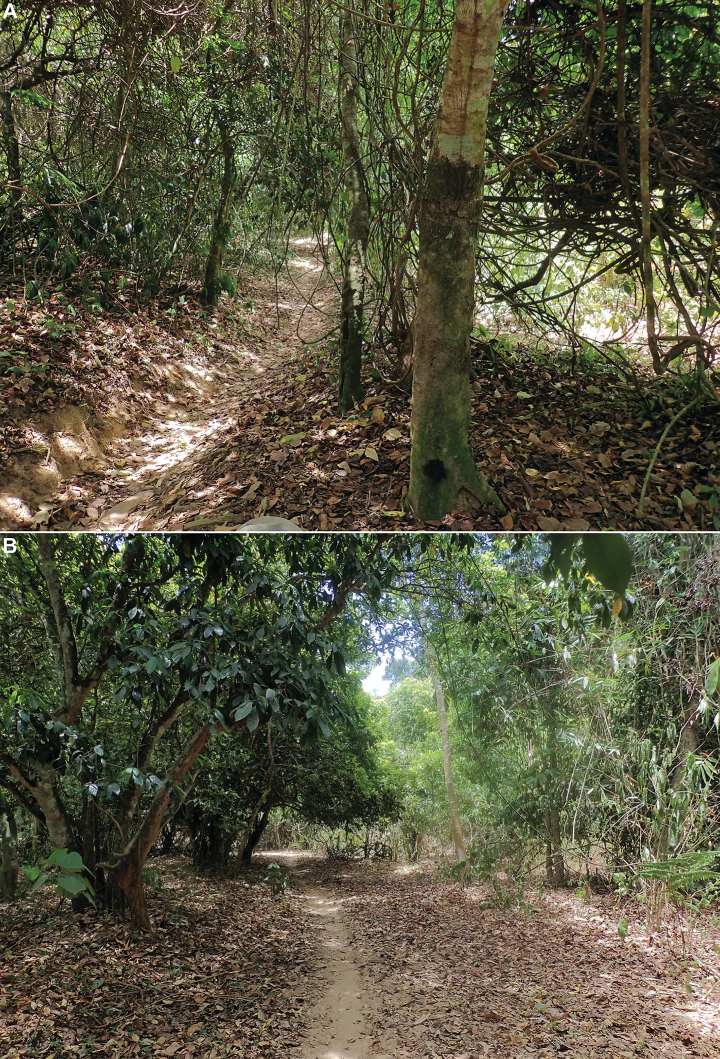
A, B. Habitat of *Gelastyrella vuquangensis* sp. nov. in Vu Quang National Park, 14 July 2023.

The specimens were found sitting on tree trunks and larger branches covered in moss and lichen (Figs 6, 7). Their colouration makes them very cryptic and difficult to spot in their habitat, and the insects seem very confident in their protection strategy, as they often will not move until touched. Despite the exceptionally dry conditions in July 2023, the species was rather common around Ke Chè station.

##### Distribution.

**Figure 8.**
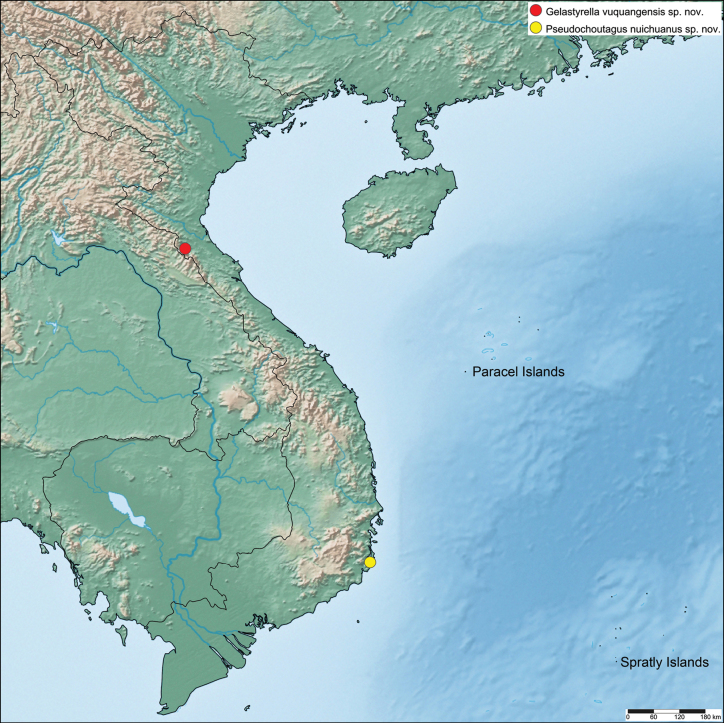
Distribution map of *Gelastyrella vuquangensis* sp. nov. and *Pseudochoutagus nuichuanus* sp. nov.

Vietnam, Ha Tinh Province, Vu Quang National Park (Fig. 8).

#### ﻿Genus Pseudochoutagus

Taxon classificationAnimaliaHemipteraIssidae

Che, Zhang & Wang, 2011

27545F35-C204-5670-8F4E-DEC043D73317

Pseudochoutagus Che, Zhang & Wang, 2011: 63. Type species: Pseudochoutagus curvativus Che, Zhang & Wang, 2011, by original designation.

##### Diagnosis.

The genus *Pseudochoutagus* can be differentiated from all other genera of Parahiraciina by the following combination of characters (Che et al. 2011; Constant 2021):

1. head prolongated anteriorly by an elongate, straight, acutely pointed cephalic process;
2. vertex and distal half of frons with moderate median carina;
3. sides of body rounded;
4. metatibiae with seven apical spines;
5. anal tube of male spatulate and with apical margin weakly emarginate;
6. aedeagus with pair of long basiventral processes projecting cephalad then reflexed and distally sinuate;
7. pygofer subrectangular in lateral view, without posterior process.

##### Species included (distribution).

*Pseudochoutagus curvativus* Che, Zhang & Wang, 2011 (China (Hainan) – Che et al. 2011).

*Pseudochoutagus lindae* Constant & Pham, 2024 (Vietnam – Constant and Pham 2024b)

*Pseudochoutagus nuichuanus* sp. nov. (Vietnam).

*Pseudochoutagus rubens* Gnezdilov & Constant, 2012 (Vietnam – Gnezdilov and Constant 2012).

*Pseudochoutagus trungi* Constant & Pham, 2024 (Vietnam – Constant and Pham 2024b).

#### ﻿ Pseudochoutagus nuichuanus sp. nov.

Taxon classificationAnimaliaHemipteraIssidae

8840C42E-DC87-57F4-A3A8-5C9E1ACC04AC

https://zoobank.org/C7BCCA1C-357C-42E4-ABA1-375A79B8F7E2

Figs 8, 9, 10, 11, 12, 13, 14, 15

##### Type material.

***Holotype*** ♂, Vietnam • Khanh Hoa Province, Nui Chua National Park, 30 Jun.–8 Jul. 2025; 11°46'28"N, 109°11'55"E; 50–700m; J. Constant, J. Bresseel, L. Semeraro, Trung Thanh Vu leg.; VNMN-E.: 000.016.110; VNMN.

***Paratypes***, Vietnam • 2 ♀♀; same data as for holotype; VNMN-E.: 000.016.111-112; VNMN • 1 ♂; same data as for holotype; I.G.: 35028; RBINS • 3 ♀♀; Khanh Hoa province, Nui Chua National Park, 3–6 Oct. 2024; 11°46'28"N, 109°11'55"E; 50-700m elev.; J. Constant, J. Bresseel, L. Semeraro, Hoai T.T. Nguyen leg.; I.G.: 34893; RBINS.

##### Diagnosis.

The species is closest externally to *P. rubens* Gnezdilov & Constant, 2012 (see illustrations in Constant 2021: fig. 5), and *P. trungi* Constant & Pham, 2024 (see illustrations in Constant and Pham 2024b: figs 28–30), but *P. rubens* differs by the dark, reddish brown body (variegated brown washed with yellowish in *P. nuichuanus* sp. nov.), and both *P. rubens* and *P. trungi* differ by the more robust, relatively shorter and very weakly curved cephalic process (process more slender, relatively longer and distinctly upcurved in distal portion in *P. nuichuanus* sp. nov.).

##### Description.

***Measurements and ratios***: LT: ♂ (n = 2): 9.4 mm; ♀ (n = 5): 10.3 mm (10.2–10.5). LT/BB = 2.5; LTg/BTg = 2.0; LW/BW = 1.5; LV/BV = 2.8; LF/BF = 2.6.

**Figure 9.**
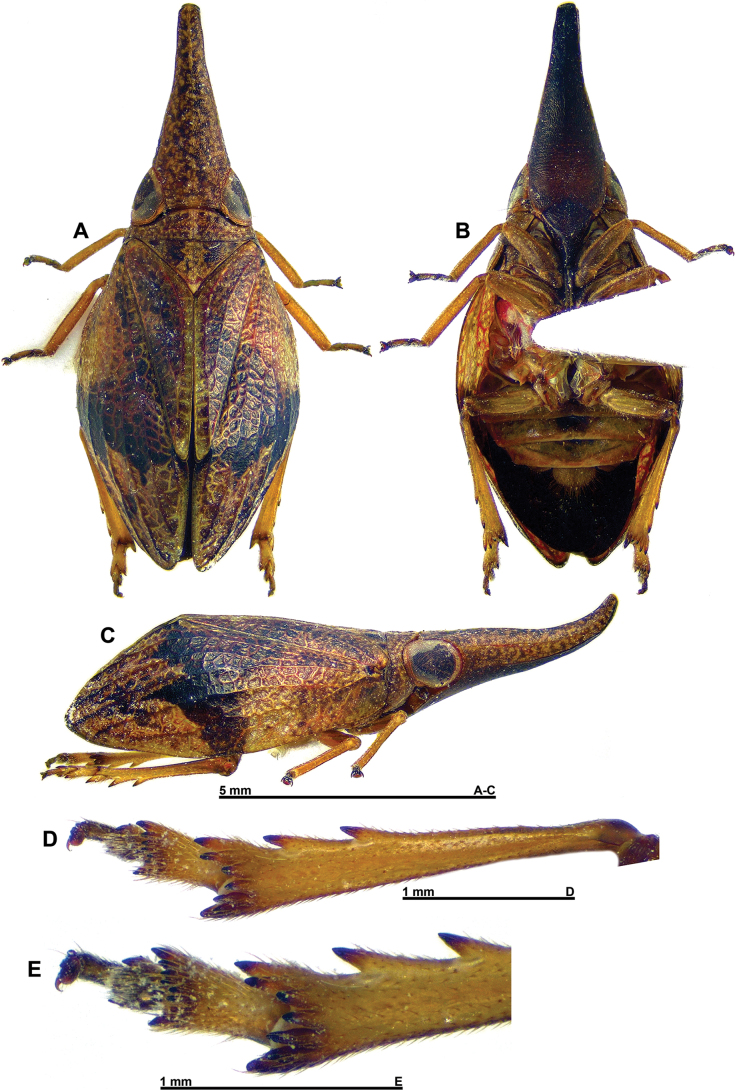
*Pseudochoutagus nuichuanus* sp. nov., paratype ♀ (RBINS). A. Habitus, dorsal view; B. Habitus, ventral view; C. Habitus, lateral view; D. Metatibia and metatarsus, ventral view; E. Distal portion of metatibia and metatarsus, ventral view.

**Figure 10.**
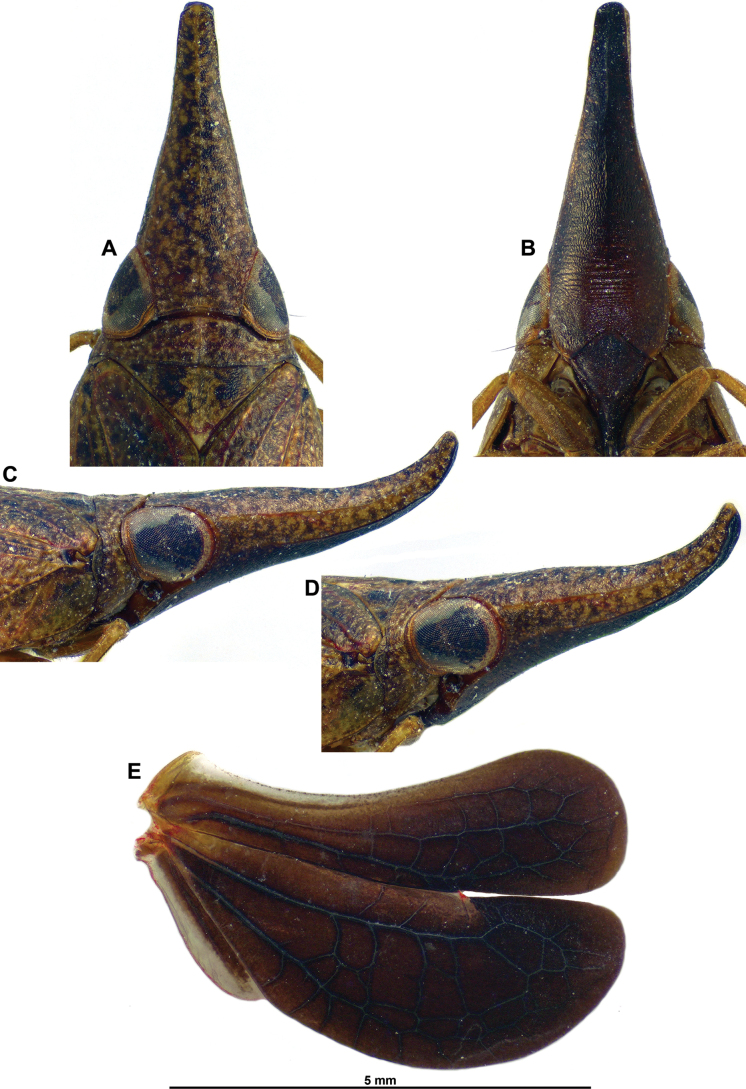
*Pseudochoutagus nuichuanus* sp. nov., paratype ♀ (RBINS). A. Head and thorax, dorsal view; B. Frons, perpendicular view; C. Head and thorax, lateral view; D. Head and thorax, anterolateral view; E. Right hind wing.

***Head*** (Figs 9A–C, 10A–D): strongly elongated anteriorly in a cephalic process slightly sinuate with distal portion upcurved (in lateral view); vertex variegated brown; distinctly longer in midline than broad before eyes (about 2.8 times), widening from base to anterior margin of eyes, tapering beyond eyes to narrowly rounded apex, median and lateral carinae distinct but rather weak; posterior margin moderately incurved. Side of head brown but coloured as vertex on sides of cephalic process. Frons entirely blackish brown, distinctly darker than sides of head and vertex, slightly coriaceous; lateral carinae reaching apex, median carina distinct in distal 2/3; elongate, widest slightly anterior to eyes, roundly tapering towards clypeal suture; clypeal suture distinctly rounded. Clypeus black-brown, weakly elevated medially in distal portion. Labium brown with last segment longer than broad, and shorter than penultimate. Antennae dark brown; scape short, ring-shaped; pedicel bulbous.

***Thorax*** (Figs 9A, C, 10A–D): pronotum coloured as vertex; about 2/3 the length of mesonotum in midline; anterior margin weakly carinate, sinuate and moderately protruding anteriorly between eyes, with peridiscal carinae indistinct; posterior margin nearly straight; median carina obsolete; weak impressed point on each side of median line; weak blunt, tubercles along anterior and posterior margins; paranotal lobes (lateral view) moderately broad, with posteroventral angle rather narrowly rounded. Mesonotum coloured like vertex with longitudinal carinae very weak and blunt; 2 weakly impressed points on disc; tip of scutellum pale yellow. Tegulae brown.

***Tegmina*** (Figs 9A–C, 14): variegated brown, washed with yellowish in basal portion, irregular black transverse band just beyond midlength; at about basal 1/3, more or less distinct transverse band of white wax, apical 1/3 covered in white wax, basal 1/3 with scattered small points of white wax; veins concolorous except in distal 1/3, where dark brown; subcoriaceous with longitudinal veins distinct (elevated in distal 1/3 of tegmen) and with dense reticulum of veinlets; shape elongate and convex with sides broadly rounded, widest around half-length, about twice as long, as broad; narrowly rounded apically. Postclaval margin widely rounded and notched at apex of clavus. Clavus closed, reaching about 3/5 of tegmen.

Venation: ScP+R forking close to base after short common stem, ScP+RA and RP single, running more or less parallel to costal margin in a large basal portion; MP forked slightly before basal 1/3, resulting veins unforked, running more or less parallel; CuA weakly diverging from claval joint; Pcu and A_1_ fused at distal 1/4 of clavus, resulting Pcu+A_1_ reaching claval joint before apex of clavus; dense reticulum of veinlets, especially after basal 1/3; veinlets distinctly raised in basal 2/3 (adding to coriaceous aspect).

***Hind wings*** (Fig. 10E): black-brown, slightly paler along costal margin in basal half; venation concolorous (slightly darker); wing broader than tegmen and deeply bilobed at CuP; costal margin sinuate; CuP-Pcu-A_1_ lobe broadly rounded along postclaval margin and about 1.3 times as wide as Sc-R-MP-CuA lobe, both about the same length and apically rounded; A_2_ lobe moderately reduced and narrow.

Venation: longitudinal veins ScP-R-MP-Cu well distinct; Pcu and A_1_ separated; numerous cross-veins; A_2_ rather weak.

***Legs*** (Fig. 9A–E): moderately elongate, slender; yellow-brown with pro- and mesotarsi and distal portion of metafemora, brown; spines of hind legs apically black; femora wider than corresponding tibiae; metatibiae with 2 lateral spines in distal half and 7 apical spines. Metatibiotarsal formula: (2) 7 / 9–10 / 2.

***Abdomen*** (Fig. 9B): yellowish brown with dark brown mediobasal marking (stronger on basal segments).

**Figure 11.**
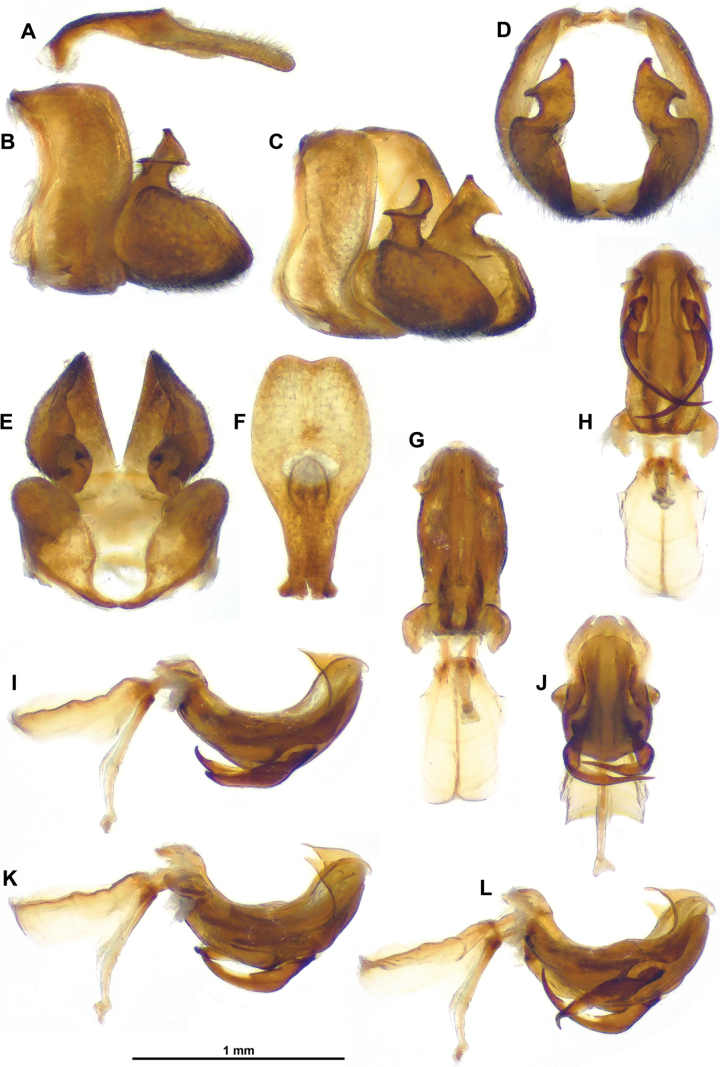
*Pseudochoutagus nuichuanus* sp. nov., holotype ♂ (VNMN). A. Anal tube, lateral view; B–E. Pygofer and gonostyli; B. Lateral view; C. Posterolateral view; D. Caudal view; E. Dorsal view; F. Anal tube, dorsal view; G–L. Aedeagus; G. Dorsal view; H. Ventral view; I. Lateral view; J. Caudal view; K. Laterodorsal view; L. Lateroventral view.

**Figure 12.**
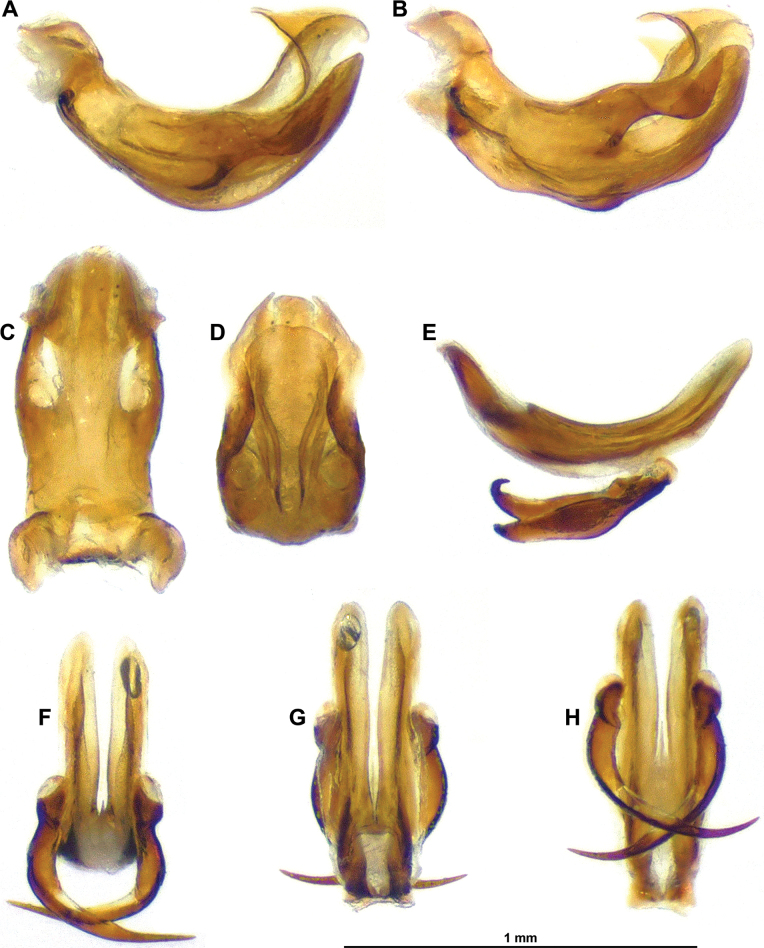
*Pseudochoutagus nuichuanus* sp. nov., holotype ♂ (VNMN). A–D. Periandrium; A. Lateral view; B. Lateroventral view; C. Dorsal view; D. Caudal view; E–H. Aedeagus ss; E. Lateral view; F. Caudal view; G. Dorsal view; H. Ventral view.

***Terminalia*** ♂: (Figs 11, 12): pygofer (Fig. 11B–E) about 2.2 × as high as long in lateral view, with anterior and posterior margins subparallel, sinuate; subcircular in caudal view; posterior margin with posterodorsal angle nearly right and dorsal margin straight; in dorsal view, posterior margin very deeply and roundly excavate. Gonostyli (Fig. 11B–E) (in lateral view) longer than high (without dorsal capitulum), tapering posteriorly in a distinct lobe narrowly rounded apically and concave; capitulum with wide neck, directed posterodorsad, with distal portion obliquely concave, smooth and ended in a tooth curved laterodorsad and with outer margin under lateral laminate projection, strongly concave in caudal view. Aedeagus (Figs 11G–L, 12) symmetrical, distinctly curved dorsad (in lateral view), longer and wider than ventral lobe of periandrium; dorsal lobe of periandrium with narrow, tapering median process and with pair of posterolateral pointed processes recurved cephalad, lateroventral margin distinctly sinuate and partly covered by ventral lobe, together forming an incomplete tube around aedeagus ss; ventral lobe of periandrium evenly widening posterior to lateroventral processes of aedeagus, with apical margin rounded; aedeagus sensu stricto elongate, bifid, with pair of massive, elongate lateroventral processes evenly curved mesocephalad, originating slightly beyond half length, more or less regularly tapering to narrowly pointed apex not reaching base of aedeagus. Connective elongate, weakly curved, with massive tectiductus with dorsal crista. Anal tube (Fig. 11A, F) dorsoventrally flattened, in dorsal view spatulate with apical margin roundly emarginate (bisinuate), about 2.0 × as long in midline, a wide; basal portion narrow, widening to strong sinuation at level of anal opening; distal portion weakly, roundly tapering to lateroposterior rounded angles; in lateral view, strongly curved near base, then nearly straight; anal opening at about midlength.

**Figure 13.**
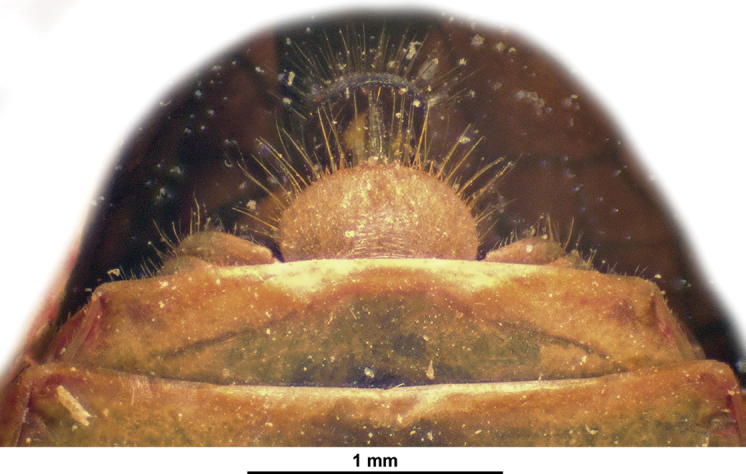
*Pseudochoutagus nuichuanus* sp. nov., paratype ♀ (RBINS), terminalia.

***Terminalia*** ♀ (Fig. 13): hind margin of sternum VII with massive, rather short, apically rounded median process.

##### Etymology.

The species epithet *nuichuanus* refers to Nui Chua National Park in Khanh Hoa Province, where the new species was discovered.

##### Biology.

**Figure 14.**
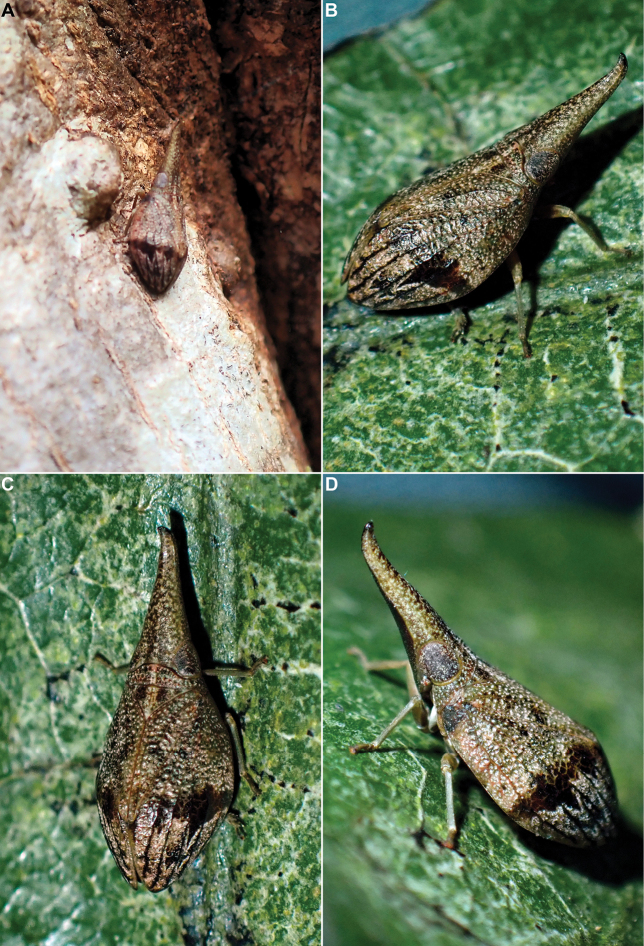
*Pseudochoutagus nuichuanus* sp. nov., live specimen in Nui Chua National Park, 4 October 2024. A. In situ on tree trunk; B–D. Photographed in cage.

**Figure 15.**
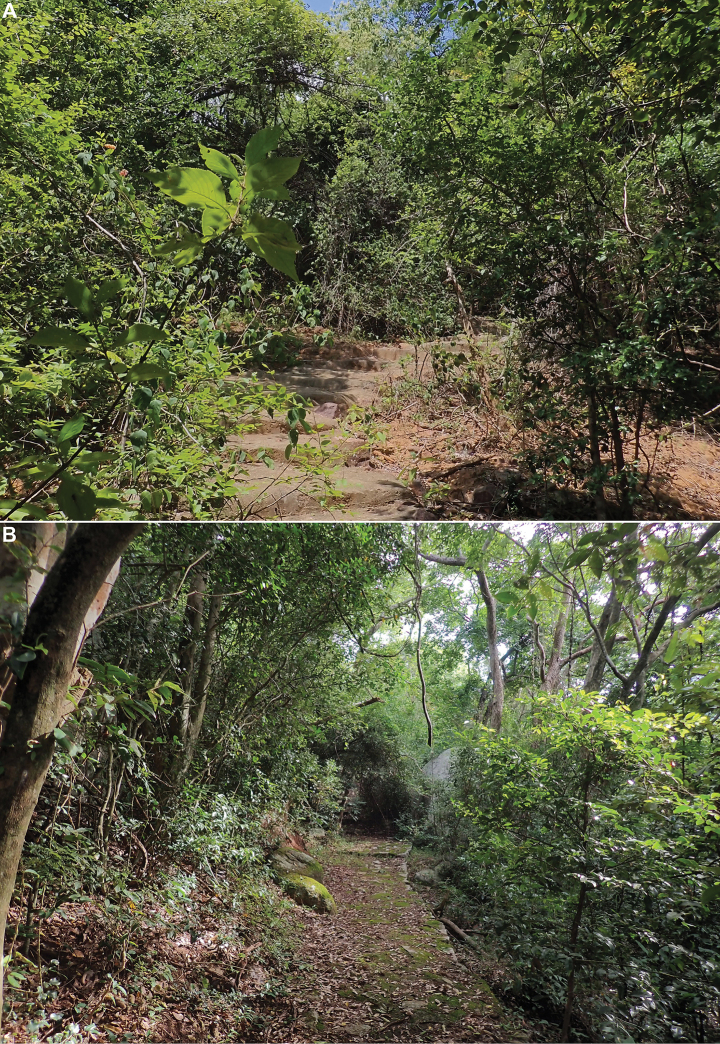
Habitat of *Pseudochoutagus nuichuanus* sp. nov. in Nui Chua National Park, 4 October 2024. A. Dry area; B. Evergreen forest.

One specimen was found sitting on a tree trunk in a rather dry area (Figs 14A, 15A), and the others were swept from dense bushes (Fig. 15B) in subtropical evergreen forest. When disturbed, they tend to run to escape, to the other side of the trunk they sit on, rather than to jump away.

##### Distribution.

Vietnam, Binh Thuan Province, Nui Chua National Park (Fig. 8).

## Supplementary Material

XML Treatment for Parahiraciina

XML Treatment for Gelastyrella

XML Treatment for Gelastyrella vuquangensis

XML Treatment for Pseudochoutagus

XML Treatment for Pseudochoutagus nuichuanus

## ﻿Additional information

### Use of AI

No use of AI was reported.

### Author ORCIDs

Jérôme Constant https://orcid.org/0000-0003-0254-0863

Thai-Hong Pham https://orcid.org/0000-0002-4763-3679

Hoai Thu Thi Nguyen https://orcid.org/0009-0004-9130-2940

### Data availability

All of the data that support the findings of this study are available in the main text.
